# Whole-Brain Radiation Therapy With Simultaneous Integrated Boost Versus Whole-Brain Radiation Therapy Plus Stereotactic Radiosurgery for the Treatment of Brain Metastasis From Lung Cancer

**DOI:** 10.3389/fonc.2021.631422

**Published:** 2021-03-05

**Authors:** Binwei Lin, Dan Huang, Huan Du, Jinjia Fan, Yu Zhang, Gang Feng, Feng Gao, Xiao Bo Du

**Affiliations:** ^1^ Department of Oncology, Mian Yang Central Hospital, Mianyang, China; ^2^ Radiology Department, Mian Yang Central Hospital, Mianyang, China; ^3^ Department of Oncology, Affiliated Hospital of North Sichuan Medical College, Nan Chong, China

**Keywords:** brain metastasis, whole-brain radiation therapy, simultaneous integrated boost, stereotactic radiosurgery, prognosis

## Abstract

Radiotherapy is one of the most important treatments for brain metastasis (BM). This study aimed to assess whether whole-brain radiation therapy (WBRT) with simultaneous integrated boost (SIB) provided any therapeutic benefit compared to WBRT followed by stereotactic radiosurgery (SRS). Seventy-two consecutive cases of lung cancer with BM treated from January 2014 to June 2020 were analyzed retrospectively. Thirty-seven patients were treated with WBRT (30 Gy in 10 fractions) and SIB (45 Gy in 10 fractions), and 35 patients were treated with WBRT (30 Gy in ten fractions) followed by SRS (16–24 Gy according to the maximum tumor diameter). The primary endpoint was intracranial progression-free survival (PFS). The secondary endpoints were intracranial objective response (partial and complete responses), pattern of intracranial progression, overall survival (OS), and toxicity. The WBRT + SIB group had a significantly longer median intracranial PFS (9.1 vs. 5.9 months, P = 0.001) than the WBRT + SRS group. The intracranial objective response rate was 67.6% and 62.9% in the WBRT + SIB and in WBRT + SRS groups, respectively (P = 0.675). The incidence of progression outside the P-GTV in the WBRT + SIB group was significantly lower than that in the WBRT + SRS group (39.4% vs. 75.0%, P = 0.004). The median OS was 24.3 and 20.3 months in the WBRT + SIB and WBRT + SRS groups, respectively (P = 0.205). There was no significant difference in the incidence of grade 3 or worse adverse reactions between the two groups. Compared to treatment with WBRT + SRS, that with WBRT + SIB for BM appeared to contribute to local control.

## Introduction

Lung cancer is the most common cause of cancer-related death worldwide (1), and up to 30% of lung cancer cases will develop brain metastasis (BM) during the course of the disease (2, 3). The prognosis of BM is very poor, and the median survival time is only 1–2 months when only corticosteroid hormones are used (4). Currently, the National Comprehensive Cancer Network guidelines recommend resection or stereotactic radiosurgery (SRS) for patients with limited BM. Moreover, whole-brain radiation therapy (WBRT) avoiding the hippocampus is an important strategy, especially for patients with unresectable tumors or those in whom SRS is not indicated (5–8). In some cancer centers, there are no criteria for administering SRS and WBRT is used an alternative strategy for patients with limited BM. However, WBRT alone only extends the median survival time by up to 6 months (9, 10). WBRT plus in-field radiotherapy boost is an effective strategy for improving the intracranial control rate (ICR) and selecting patients who could experience significant survival benefits (11–17). WBRT followed by SRS (WBRT + SRS) and WBRT combined with simultaneous integrated boost (SIB) are the two main boost schemes for BM. Andrews (12) and Aoyama (17) reported that WBRT + SRS had a better ICR and significantly prolonged the survival for patients with BM or graded prognostic assessment scores of 2.5–4.0 compared to WBRT alone.

WBRT + SIB is another boost scheme for BM. Compared with SRS, SIB is more convenient since only a single radiotherapy plan is needed, reirradiation is easier as the dose to the organs at risk is known, and radiation damage to normal brain tissue is reduced in the treatment of tumors with large diameters for which SRS is not indicated (18).

A dosimetric study showed that WBRT combined with SIB (WBRT + SIB) could satisfy the target dose and protect the surrounding normal tissues such as the hippocampus (19). Several studies have also reported that WBRT + SIB significantly improves ICR and survival compared to WBRT alone (14, 16, 20). However, it is unclear whether WBRT + SIB can improve efficacy and reduce toxicity compared to WBRT + SRS. Therefore, this retrospective analysis aimed to determine whether WBRT + SIB provided any therapeutic benefit compared to WBRT + SRS.

## Materials and Methods

### Patients

This single-center retrospective study was approved by the institutional ethics committee (No: P2019012). Consecutive patients with BM who received radiotherapy at our hospital from January 2014 to June 2020 were included. The inclusion criteria were as follows: (1) primary lung cancer confirmed pathologically and BM confirmed on brain magnetic resonance imaging (MRI), (2) patient age ≥18 years, (3) Karnofsky Performance Status (KPS) score ≥70, and (4) radiotherapy including WBRT + SIB or WBRT + SRS. The exclusion criteria included the following: (1) resection of intracranial lesions before radiotherapy, (2) prophylactic cranial irradiation for small-cell lung cancer, and (3) previous treatment with intracranial radiotherapy.

Clinical data on age, sex, number of BMs, maximum diameter of BM, pathological type of tumors, status of extracranial metastases, recursive partition analysis (RPA) classification, KPS score before radiotherapy, best response during follow-up, whether chemotherapy or targeted therapy was administered after radiotherapy, dosage of radiotherapy, date of radiotherapy, date of intracranial progression, pattern of intracranial progression, date of death or final follow-up visit, and toxic reactions associated with radiotherapy were recorded.

### Radiotherapy Strategy

Radiotherapy was delivered by 6 MV photon beam linear accelerators. The target delineation criteria for the WBRT + SIB and WBRT + SRS groups were the same—the gross tumor volume (GTV) encompassed contrast-enhanced BM on T1-weighted MRI. The P-GTV was defined as a 3-mm margin to the GTV, the clinical target volume (CTV) encompassed the whole brain, and the planning target volume (PTV) was defined as a 3-mm margin to the CTV. For patients who underwent WBRT + SIB, the prescribed dose to the PTV was 30 Gy in 10 fractions and the simultaneous boost to the P-GTV was 15 Gy in 10 fractions (P-GTV: 45 Gy in 10 fractions) once a day (Monday to Friday). For patients who underwent WBRT + SRS, the prescribed dose to the PTV was 30 Gy in 10 fractions once a day (Monday to Friday), and SRS was performed 1 day after the end of WBRT. The prescribed dose for SRS varied according to the tumor diameter; for tumors measuring ≤2 cm and >2 cm in diameter, the prescribed dose was 18–24 Gy in 1 faction and 16 Gy in 1 faction, respectively. The distance from the isodose curve of 37.5 Gy to the boundary of the P-GTV at the maximum tumor diameter, integral dose to the P-GTV, and 50% and 100% volume (V50% and V100%, respectively) of the isodose line of the P-GTV were recorded. V50%/V100% was used to calculate the Gradient indices (GIs) (21).

### Follow-up

Clinical evaluations and brain MRI were performed at 1-month intervals up to 3 months after radiotherapy. The responses were evaluated by experienced radiologists according to the RECIST 1.1 criteria. Toxicities associated with radiotherapy were evaluated according to the RTOG central nervous system toxicity criteria.

### Study Endpoints

The primary endpoint was the median intracranial progression-free survival (PFS). The secondary endpoints were intracranial objective response (partial and complete responses), pattern of intracranial progression, overall survival (OS), and toxicity associated with radiotherapy. The intracranial PFS was defined as the time from radiotherapy to intracranial progression or death. The OS was defined as the time from radiotherapy to death or the last follow-up. Progression was defined as a >20% increase in the diameter of BM or the presence of new intracranial BM on brain MRI.

### Statistical Analysis

The Kaplan–Meier method was used to analyze intracranial PFS and OS of the two radiotherapy methods, and the log-rank test was used to compare the difference between the two groups. The chi-square test was used to compare the differences in clinical factors, objective response rate, and incidence of toxicity between the two groups. Univariate analysis was performed using the log-rank test. The clinical factors with P < 0.1 in univariate analysis were included in multivariate analysis. The Cox regression model was used to analyze the prognostic factors of intracranial PFS. SPSS 19.0 software (IBM, Chicago, IL, USA) was used for statistical analyses. P < 0.05 was considered statistically significant.

## Results

### Patients

From January 2014 to June 2020, 167 lung cancer patients with BM received radiotherapy. Among these patients, 79 patients only received WBRT because the number of BMs was >10, three patients underwent surgical resection of BM before radiotherapy, one patient underwent reirradiation, and 12 patients had missing follow-up data. Hence, only 72 patients with BM (37 patients treated with WBRT + SIB and 35 patients treated with WBRT + SRS) were included in the study. The clinical characteristics of the two groups are shown in **Table 1**. The proportion of BM with a diameter of ≤3 cm was higher in the WBRT + SRS group than in the WBRT + SIB group, but the difference was not statistically significant (P = 0.057). There was no significant difference in other clinical features between the two groups.

**Table 1:** Patients characteristics.

Characteristics	WBRT + SIB(n = 37)	WBRT + SRS(n = 35)	P
Median age at BM diagnosis (range) y	58(30–85)	57(41–74)	0.192
<65	24(64.9%)	28(80.0%)	
≥65	13(35.1%)	7(20.0%)	
Male	24(64.9%)	18(51.4%)	0.339
Number of BM			0.812
1	15(40.5%)	13(37.1%)	
≥2	22(59.5%)	22(62.9%)	
Diameter of largest BM(cm)			
mean(sd)	2.8(1.6)	1.8(1.2)	
Median (range)	2.2(0.7–8.4)	1.5(0.5–5.7)	0.057
≤3cm	24(64.9%)	30(85.7%)	
>3cm	13(35.1%)	5(14.3%)	
Extracranial disease			0.249
Stable	10(27.0%)	5(14.3%)	
Active	27(73.0%)	30(85.7%)	
RPA			1.000
1	4(10.8%)	4(11.4%)	
2	33(89.5%)	31(88.6%)	
			
Histological status			0.368
Adenocarcinoma	28(75.7%)	22(62.9%)	
Small cell	4(10.8%)	8(22.8%)	
Squamous cell	5(13.5%)	5(14.3%)	
KPS			0.350
80	36(97.3%)	32(91.4%)	
70	1(2.7%)	3(8.6%)	
Dose of SRS(Gy)			
24	–	2	
20	–	1	
18	–	9	
16	–	23	
Chemotherapy after RT			0.487
Yes	18(48.6%)	14(40.0%)	
No	19(51.4%)	21(60.0%)	
Target therapy after RT			0.641
Yes	17(45.9%)	14(40.0%)	
No	20(54.1%)	21(60.0%)	

WBRT, whole brain radiation therapy; SIB, simultaneous integrated boost; SRS, stereotactic radiosurgery; BM, brain metastases; RPA, recursive partition analysis; KPS, karnofsky performance status; RT, radiotherapy.

The median follow-up time was 18.4 months (range, 2.3–78 months). Five patients were lost to follow-up, and the follow-up rate was 93.1%.

### Best Overall Response, Pattern of Intracranial Progression, and Dosimetric Parameters

The WBRT + SIB and WBRT + SRS groups had a similar objective response rate (67.6% vs. 62.9%, P = 0.675, **Table 2**). At the last follow-up, 33 patients in the WBRT + SIB group and 32 patients in the WBRT + SRS group had intracranial progression. The progression rate of the P-GTV in the WBRT + SIB group was significantly higher than that in the WBRT + SRS group (60.6%% vs. 25.0%, P = 0.004). Compared to the WBRT + SIB group, the WBRT + SRS group received a significantly higher integral dose to the P-GTV (47.22 vs. 45.19 Gy, P < 0.001), had significantly lower GIs (2.72 vs. 3.51, P < 0.001), and had a shorter distance from the isodose curve of 37.5 Gy to the boundary of P-GTV (1.20 vs. 2.09 cm, P < 0.001, **Table 3**).

**Table 2:** Overall response according to RECIST1.1 criteria.

	WBRT+SIB	WBRT+SRS	P
Objective response	25(67.6%)	22(62.9%)	0.675
Best overall response			
Complete response	3(8.1%)	2(5.7%)	
Partial response	22(59.5%)	20(57.1%)	
Stable disease	4(10.8%)	7(20.0%)	
Progressive disease	8(21.6%)	6(17.2%)	

WBRT, whole brain radiation therapy; SIB, simultaneous integrated boost; SRS, stereotactic radiosurgery.

**Table 3:** Pattern of intracranial progression and dosimetric parameters.

	WBRT + SIB (n = 37)	WBRT + SRS (n = 35)	P
Total of progressions	33	32	
Within the P-GTV	20(60.6%)	8(25.0%)	0.004
Out of the P-GTV	13(39.4%)	24(75.0%)	
Dosimetric parameters			
Distance(37.5Gy – P-GTV)	2.09 ± 0.57	1.2 ± 0.29	<0.001
D95%(P-GTV)	45.19 ± 0.10	47.22 ± 2.25	<0.001
GI	3.51 ± 0.52	2.72 ± 0.28	<0.001

P-GTV = 3 mm margin to gross tumor volume. WBRT, whole brain radiation therapy; SIB, simultaneous integrated boost; SRS, stereotactic radiosurgery.

Distance (37.5Gy – P-GTV), distance from the isodose curve of 37.5 Gy to the boundary of P-GTV at the maximum diameter of the tumor;

GI, gradient index.

### Intracranial PFS and OS

**Figure 1:**
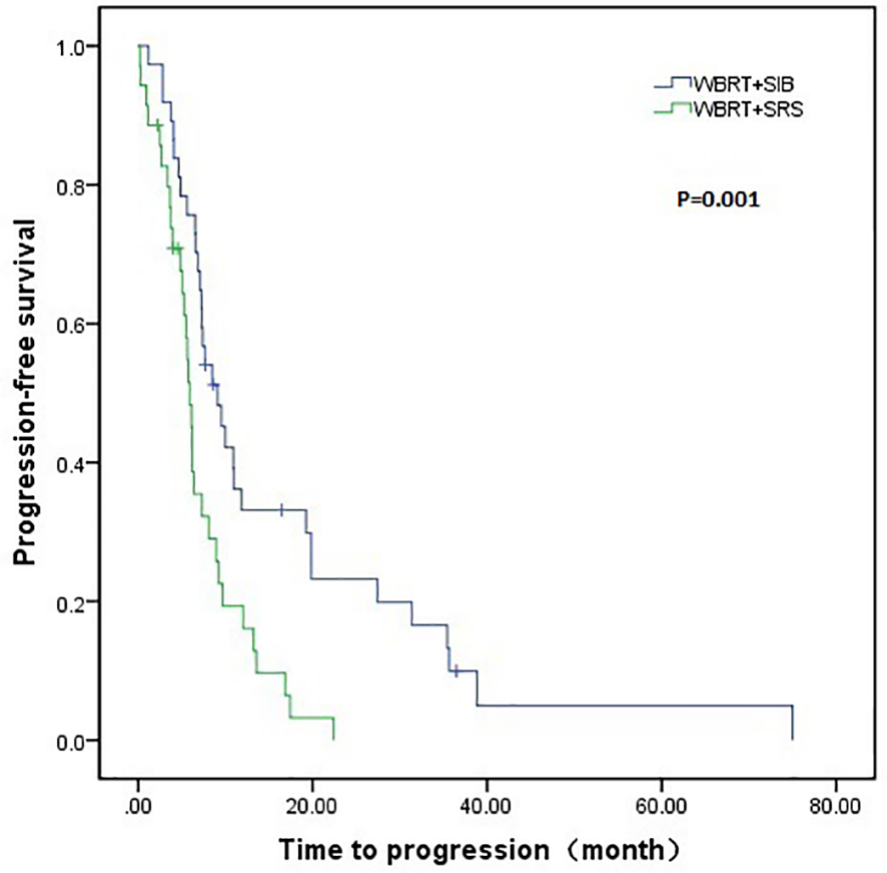
Comparison of intracranial progression-free survival between WBRT + SIB and WBRT + SRS.

**Figure 2:**
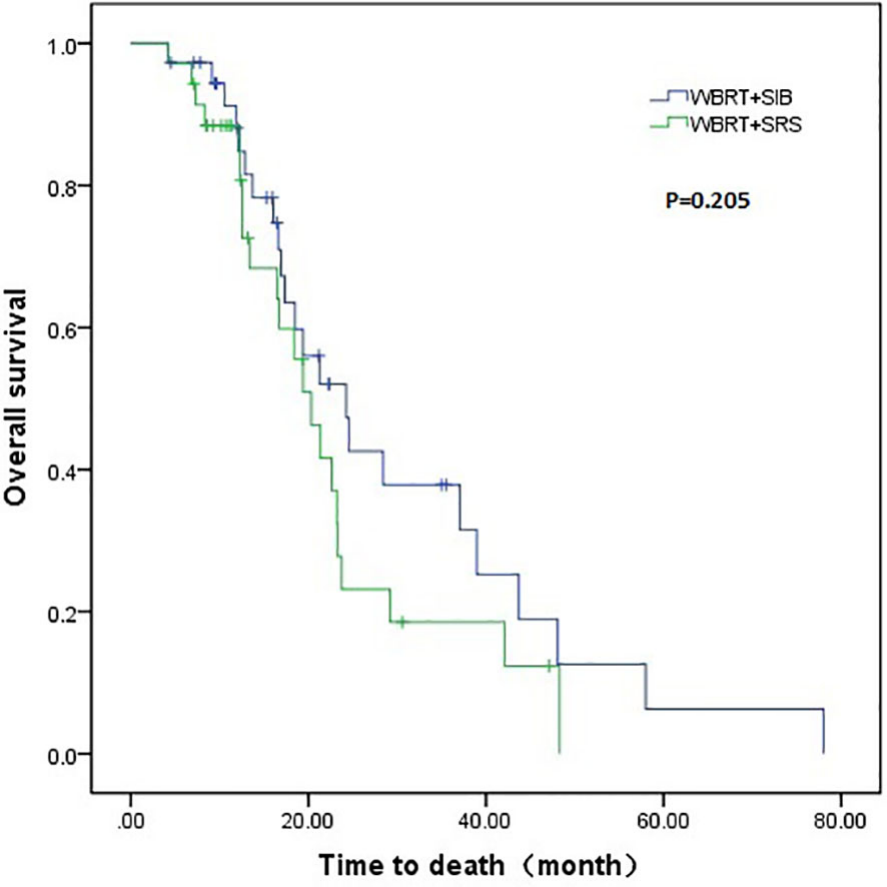
Comparison of overall survival between WBRT + SIB and WBRT + SRS.

The WBRT + SIB group had a longer median intracranial PFS than the WBRT + SRS group (9.1 vs. 5.9 months, P = 0.001). Furthermore, 7 (18.9%) patients in the WBRT + SIB group and no patients in the WBRT + SRS group had a disease control status at 2 years after radiotherapy (**Figure 1**). The median OS was similar between the WBRT + SIB and WBRT + SRS groups (24.3 vs. 20.3 months, P = 0.205, **Figure 2**).

### Predictive Factors of Intracranial PFS

Univariate analysis showed that pathological type of tumors and the radiotherapy method significantly affected the intracranial PFS (**Table 4**). The radiotherapy method, sex, pathological type of tumors, and administration of targeted therapy were included in the Cox regression model. The Cox regression model analysis showed that in the WBRT + SIB group, targeted therapy after radiotherapy and male sex were associated with a significantly longer intracranial PFS (**Table 5**).

**Table 4:** Survival-related factors on intracranial PFS in univariate analysis.

Variable	NO. of Participants	Median PFS (month)	Log-Rank P Value
Treatment group			0.001*
WBRT+SIB	37	9.10	
WBRT+SRS	35	5.90	
Age			0.751
<65	52	7.38	
≥65	20	5.50	
Sex			0.077
Male	42	9.10	
Female	30	5.75	
No. of BM			0.545
1	28	6.53	
≥2	44	8.10	
Diameter of largest BM			0.564
≤3cm	54	6.53	
>3cm	18	7.30	
Histological status			0.014*
Adenocarcinoma	50	8.93	
small cell lung cancer	12	3.93	
Squamous cell carcinoma	10	6.85	
RPA			0.173
1	8	6.15	
2	64	7.25	
Extracranial disease			0.169
Stable	57	7.25	
Active	15	7.30	
KPS			0.137
70–80	68	7.30	
90–100	4	3.93	
Chemotherapy after RT			
Yes	32	7.38	0.593
No	40	7.10	
Target therapy after RT			
Yes	31	8.50	0.069
No	41	6.85	

WBRT, whole brain radiation therapy; SIB, simultaneous integrated boost; SRS, stereotactic radiosurgery; BM, brain metastases; RPA, recursive partition analysis; KPS, karnofsky performance status; RT, radiotherapy; PFS, progression-free survival.

*p < 0.05 was considered significant.

**Table 5:** Survival-related factors on intracranial PFS in multivariate analysis.

Variable	HR (95% CI)	P value
WBRT + SIB	2.31(1.34–3.98)	0.003*
Female	0.56(0.33–0.95)	0.031*
Histological status		0.442
Adenocarcinoma	1.68(0.69–4.09)	
Squamous cell carcinoma	1.11(0.48–2.53)	
Target therapy after RT	2.89(1.45–5.75)	0.003*

WBRT, whole brain radiation therapy; SIB, simultaneous integrated boost; RT, radiotherapy.

*p < 0.05 was considered significant.

### Stratified Analysis

**Figure 3:**
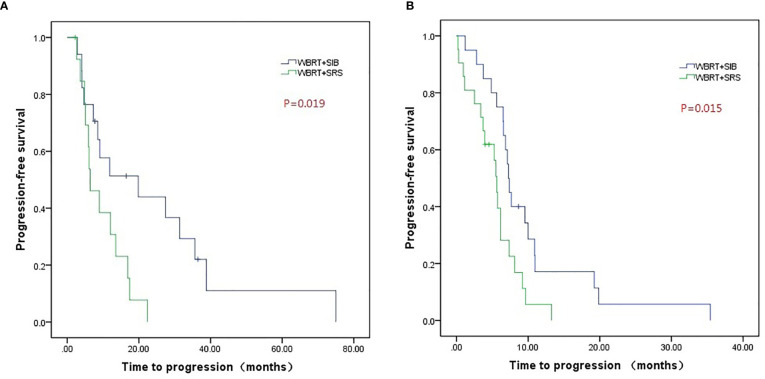
Compared intracranial progression-free survival of WBRT + SRS and WBRT + SIB in patients with **(A)** or without targeted therapy **(B)**.

**Figure 4:**
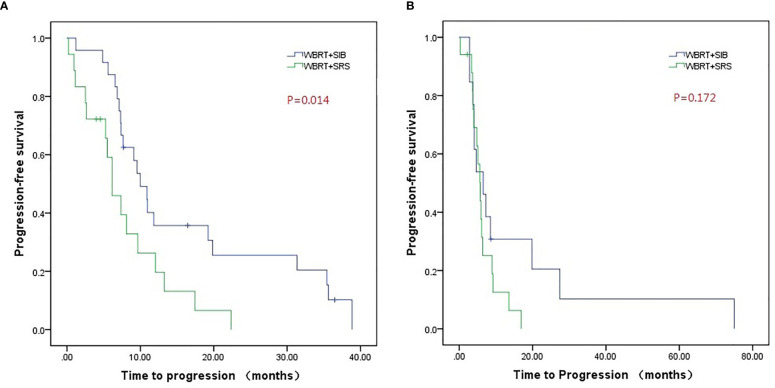
Compared intracranial progression-free survival of WBRT + SRS and WBRT + SIB in male **(A)** or female **(B)** patients.

**Figure 5:**
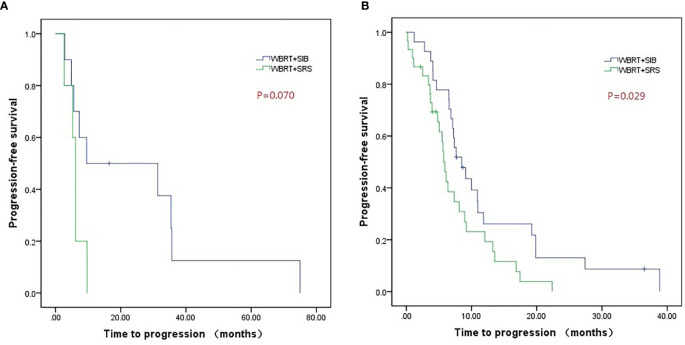
Compared intracranial progression-free survival of WBRT + SRS and WBRT + SIB in patients with stable extracranial disease **(A)** or active extracranial disease **(B)**.

Compared to WBRT + SRS, WBRT + SIB significantly improved the median intracranial PFS in patients with (19.8 vs. 6.4 months, P = 0.019, **Figure 3A**) or without targeted therapy (7.2 months vs. 5.6 months, P = 0.015, **Figure 3B**), male patients (10.0 vs. 6.2 months, P = 0.014, **Figure 4A**), and patients with active extracranial disease (8.5 vs. 5.9 months, P = 0.029, **Figure 5B**). WBRT + SIB had similar median intracranial PFS when compared to WBRT + SRS for female patients (6.5 months vs. 5.7 months, P = 0.172, **Figure 4B**), and patients with stable extracranial disease (9.5 months vs. 6.1 months, P = 0.070, **Figure 5A**).

### Toxicity

The main toxicities associated with radiotherapy were central nervous system symptoms, nausea, vomiting, and headache. In the WBRT + SIB and WBRT + SRS groups, the incidence of grade 3 or worse toxicity was 5.4% vs. 2.9% (P = 0.589), 8.1% vs. 5.7% (P = 0.690), and 8.1% vs. 5.7% (P = 0.690), respectively (**Table 6**).

**Table 6:** Toxicity associated with radiotherapy.

Toxicity	Grade	WBRT + SIB	WBRT + SRS
Central nervous system symptoms			
	0	21	16
	1	4	15
	2	10	3
	3	1	0
	4	1	1
Nausea/vomiting			
	0	16	13
	1	9	13
	2	9	7
	3	3	1
	4	0	1
headache			
	0	15	20
	1	11	3
	2	8	10
	3	3	2
	4	0	0

WBRT, whole brain radiation therapy; SIB, simultaneous integrated boost; SRS, stereotactic radiosurgery.

## Discussion

The main aim of this single-center retrospective study was to compare the efficacy and toxicity of WBRT + SIB and WBRT + SRS treatment for BM from lung cancer. The primary endpoint was the intracranial PFS. Our result showed that the intracranial PFS was significantly longer in the WBRT + SIB group than in the WBRT + SRS group (9.1 vs. 5.9 months, P = 0.001). Furthermore, WBRT + SIB, targeted therapy after radiotherapy, and male sex were significantly associated with a longer intracranial PFS. Ge et al. (22) reported the results of WBRT + SIB treatment for BM from lung cancer; the median intracranial PFS was 6 months, which was slightly shorter than observed in our study. However, in the study Ge et al., topotecan was administered as systemic treatment and targeted therapy was not administered in any patients. In contrast, 45.9% patients received targeted therapy in the WBRT + SRS group in our study. Therefore, the difference in baseline situations may be the cause of the longer intracranial PFS in our study. Lu (20) compared the efficacy of WBRT + SIB with that of WBRT; the median intracranial PFS in the WBRT + SIB group was 22.3 months, which was higher than that observed in our study. However, in the study by Lu, the radiation dose of WBRT was higher than that used in our study. Meanwhile, more patients received targeted therapy.

We also analyzed the progression pattern of different radiotherapy methods. The incidence of progression outside the dose boost area in the WBRT + SIB group was significantly lower than that in the WBRT + SRS group (39.4% vs. 75.0%). In a previous study, the dose outside the P-GTV in SRS dropped rapidly (23). Our dosimetric analysis also showed that the drop of the dose outside the P-GTV in the WBRT + SRS group was faster than that in the WBRT + SIB group and that the distance from the isodose curve of 37.5 Gy to the boundary of P-GTV was shorter in the WBRT + SRS group than in the WBRT + SIB group. As a result, the radiation dose outside the P-GTV in WBRT + SRS group was lower than that in the WBRT + SIB group, which may have led to a higher progression rate outside the P-GTV. The location of BM and distance between BMs may also impact these dosimetric factors, and hence, they should be considered in future studies. In our study, the incidence of progression in the dose-boost area in the WBRT + SIB group was significantly higher than that in the WBRT + SRS group (60.6% vs. 25.0%). This may be due to the higher biological effective dose (BED) to the P-GTV in the WBRT + SRS group than in the WBRT + SIB group. In the WBRT + SRS group, the BED was 80–120 Gy, while in the WBRT + SIB group, the BED was only 65.25 Gy (α/β = 10). Compared with WBRT + SIB, WBRT + SRS had better local control in the P-GTV area, but worse local control outside the P-GTV area. This may have led to a similar objective response rate between two groups. Further dose escalation studies with SIB for BM are warranted to determine if a BED of ≤100 Gy would be beneficial. Future studies must also include reports of acute and late toxicity, specifically radiation necrosis.

Our results showed that the WBRT + SIB and WBRT + SRS groups had similar OS. In our study, most patients had active extracranial tumors (73.0% in the WBRT + SIB group and 85.7% in the WBRT + SRS group). Therefore, controlling intracranial lesions alone may not prolong OS. Furthermore, some studies (4, 24) have shown that brain radiotherapy could effectively control intracranial lesions, but could not prolong the OS. Recently, Qing (25) reported that for patients with BM from lung cancer who received WBRT + SIB, the median OS was 10 months. In study by Qing, 40 Gy in 10 fractions was administered to the P-GTV, 30 Gy in 10 fractions was administered to the whole brain, and systemic treatment was not administered, which may have resulted in a shorter median OS than that observed in our study.

The major radiotherapy-related toxicities in the two groups included central nervous system injury, nausea or vomiting, and headache. There was no significant difference in the incidence of grade 3 or worse adverse reactions between the two groups.

Our study has some limitations. First, our study was a retrospective study. Second, the sample size was relatively small. Despite the nonsignificant differences, intracranial PFS was better in patients with >2 BMs, the largest lesion measuring >3 cm, RPA class 2, and extracranial disease active, which could have been caused by the small sample size. Moreover, slight imbalances in patient age, sex, tumor size, active extracranial disease, primary tumor pathology, and use of systemic therapy that did not attain statistical significance, perhaps due to the lack of statistical power, existed between two groups. To reduce the impact of the baseline characteristics, we conducted stratified analyses of three factors that had a greater impact on intracranial PFS, which may support our result on intracranial PFS. Finally, because the retrospective study did not record the cognitive function and quality of life of the patients, no corresponding data was assessed.

In conclusion, WBRT + SIB can prolong intracranial PFS compared to WBRT + SRS. There was no significant difference in the incidence of grade 3 or worse toxicities between the two radiotherapy methods. Prospective randomized controlled trials comparing WBRT + SIB and WBRT + SRS are needed to validate our study findings.

## Data Availability Statement

The original contributions presented in the study are included in the article/supplementary material. Further inquiries can be directed to the corresponding authors.

## Ethics Statement

The studies involving human participants were reviewed and approved by Ethics Committee of Mianyang Central Hospital. Written informed consent for participation was not required for this study in accordance with the national legislation and the institutional requirements.

## Author Contributions

Guarantor of integrity of the entire study: XD. Study concepts and design: XD and FG. Literature research: BL. Data collection: BL, DH, HD, JF, YZ, GF, and FG. Data analysis: BL and FG. Manuscript preparation: BL and FG. Manuscript editing: XD and FG. All authors contributed to the article and approved the submitted version.

## Conflict of Interest

The authors declare that the research was conducted in the absence of any commercial or financial relationships that could be construed as a potential conflict of interest.
